# Retraction notice: Comparative genomic analysis of Escherichia coli isolates from cases of bovine clinical mastitis identifies nine specific pathotype marker genes

**DOI:** 10.1099/mgen.0.001343

**Published:** 2024-12-19

**Authors:** The Microbiology Society

**Affiliations:** 1 Microbiology Society, 14–16 Meredith Street, London, UK

The authors, *Microbial Genomics* Editor-in-Chief and the Publisher are retracting the article ‘Comparative genomic analysis of *Escherichia coli* isolates from cases of bovine clinical mastitis identifies nine specific pathotype marker genes’ [1].

The authors alerted the journal to errors in their analysis which have been assessed to have significant impact on the conclusions presented in the article. The authors have also submitted a corrected version of the article to the journal for consideration for publication.

Further details of the issues are provided by the authors below:

In the original article Roary was used to construct separate pan-genomes for mammary pathogenic *E. coli* (MPEC) and bovine commensal isolates. We ran Roary using the command ‘roary -s *.gff’ twice, once on MPEC genome set and then again on the environmental *E. coli* genome. Running Roary twice resulted in two ‘gene_presence_absence.csv’ files that each listed all genes identified in each group of isolates. We manually compared the names of the genes between the two .csv files and this analysis resulted in the identification of nine genes that were listed as core genes in MPEC but were not found in the commensal / environmental *E. coli.* We reported these genes as MPEC marker genes. There were two problems with this analysis.

The first problem is that we used the -s option when running Roary. The manual comparison may have worked if we split paralogs, but the -s option does not split paralogs. Not splitting paralogs results in groups of genes being grouped together with similar genes, but possibly under different names. The manual analysis of genes, without split paralogs, resulted in us identifying the nine genes as being unique to MPEC even though these genes had multiple paralogs in the environmental isolates. We missed identifying these genes manually because they were grouped with similar genes under a different name.

The second problem with our analysis is in the overall way we used Roary. We should have analysed both sets of *E. coli* together using the command roary *.gff; and we should not have relied on a manual comparison of unique genes from the outputs of two separate Roary analyses.

In our revised analysis the genomes from both groups of *E. coli* are included in the same Roary analysis run, we did split the paralogues, and we verified our results using blast. We also go a step further and use a python script developed by Ola Brynildsrud called Scoary which takes the output from Roary and calculates the association between each gene and either MPEC or environmental *E. coli.* The output from Scory is a list of genes sorted by the strength of the association to the MPEC or the environmental *E. coli* group, and this was included in our new analysis to verify the Roary results. This analysis clearly identified the Fec operon as being associated with MPEC. Our new analysis correctly uses Roary and supplements the analysis using Scoary to determine that the fec genes are the only defining feature between MPEC and environmental *E. coli*.

## Abbreviations

MPEC: mammary pathogenic *E. coli*
